# Local Forms of *Vigna unguiculata*—Response to Osmotic Stress at Vegetative Growth Stage

**DOI:** 10.3390/ijms26178352

**Published:** 2025-08-28

**Authors:** Lyudmila Simova-Stoilova, Liliana Gigova, Valentin Velinov, Tsvetelina Stoilova

**Affiliations:** 1 Institute of Plant Physiology and Genetics, Bulgarian Academy of Sciences, Acad. G. Bonchev Str. Block 21, 1113 Sofia, Bulgaria; gigova01@gmail.com (L.G.); valentin.velinov82@gmail.com (V.V.); 2 Institute of Plant Genetic Resourses “Konstantin Malkov”, Agricultural Academy, Druzhba 2, 4122 Sadovo, Bulgaria; tz_st@abv.bg

**Keywords:** cowpea, landraces, drought, oxidative stress, metabolites, enzymes

## Abstract

Cowpea (*Vigna unguiculata* L. Walp.) is a species with superior tolerance to drought stress compared to other legumes. It is a promising crop with increasing importance in the face of global climate changes. Local forms, well adapted to particular agro-climatic conditions, are useful germplasm resources. Five Bulgarian cowpea landraces, which had displayed differences in osmotic stress tolerance at the germination stage, were subjected to severe stress (15% PEG 6000 in Hoagland nutrient media) during 16 days at the vegetative growth stage (plants with expanded trifoliate leaves). All local forms responded to the imposed stress by biomass and leaf area diminution, a slight increase in leaf water deficit and electrolyte leakage, proline accumulation in roots and leaves, and an increase in root starch and leaf phenol content. Roots presented more pronounced metabolic changes than leaves, including increased total antioxidant activity, phenolic and carbohydrate content, and proline accumulation. Under osmotic stress, tight control of oxidative stress and concerted upregulation of superoxide dismutase, catalase, glutathione transferase, and peroxidase activities in leaves were registered along with changes in certain specific isoforms, while glutathione reductase activity diminished. Antioxidant enzyme activities had different changes in stressed roots, compared to leaves, and among genotypes. The accession most sensitive to osmotic stress at germination presented more symptoms of oxidative stress at the vegetative growth stage.

## 1. Introduction

Legume crops are globally ranked third after the cereals and oilseeds in agricultural production, and second after the cereals as a food source [1,2]. Pulses are rich in protein, fiber, and trace elements and are a gluten-free, low-glycemic index food recommended by the FAO (Food and Agriculture Organization of the United Nations) as healthy foods that fully cover the essential protein and energy requirements [3,4]. Due to the symbiotic nitrogen fixation, legumes are indispensable in crop rotation, thus reducing the need for mineral fertilization. About half of the greenhouse gas emissions from agriculture come from nitrogen fertilizers; besides, legumes emit five to seven times less greenhouse gas per unit area compared to other crops [5,6]. In this way, legumes have a substantial impact on sustainable agriculture; they are considered key crops for attaining food security and mitigating climate change.

Cowpea (*Vigna unguiculata* L. Walp.) is a pulse crop with high nutritional properties of the seeds and excellent forage value of the straw [2]. Originating from West Africa, it has spread to Asia, America, and Europe, and nowadays cowpea is cultivated in sub-Saharan Africa, in tropical regions of Asia and America, and in the Mediterranean part of Europe [4,7]. Compared to other legume crops, cowpea tolerates drought and heat stress quite well, grows in a range of soil types, including rain-fed sandy soils poor in phosphorus and organic matter, and is a promising alternative crop to meet climate changes in temperate regions [8,9,10]. Despite the generally superior performance of cowpea under hot, dry conditions compared to other legumes, significant variations in tolerance to water stress among different cowpea genotypes have been reported [11,12,13].

Water scarcity is one of the most devastating stresses encountered by crops, especially in the arid and semi-arid agro-ecosystems, and the frequency and severity of drought episodes will increase in the frame of global climate change. In cowpea, grain and fodder yield losses as a result of drought episodes are reported to be about 62% and 56%, respectively [14]. Drought can occur at any stage of the cowpea growth cycle, and its detrimental effects depend on stress severity and duration, plant developmental stage, and the genetic potential to overcome stress [15]. Early-season drought stress can affect stand establishment; at flowering or pod-filling stages, drought usually directly affects sink-source relations and yield [14,15,16]. Water scarcity disturbs main physiological processes such as water balance, mineral nutrition, and symbiotic nitrogen fixation, photosynthesis, and respiration [17,18]. Plant adaptation to water stress includes a variety of morphological, physiological, and biochemical responses, such as deep rooting, stomata closure, inhibition of photosynthesis and rearrangement of the photosynthetic apparatus, osmotic adjustment by compatible solutes, and enhanced antioxidative protection [19].

Secondary oxidative stress often accompanies severe or prolonged primary abiotic stresses, including dehydration stress; coping with oxidative stress is an important part of stress tolerance [20]. Reactive oxygen species (ROS) such as singlet oxygen, superoxide anion, hydroxyl radical, and hydrogen peroxide are partially reduced forms of molecular oxygen, necessary for the normal metabolic processes but potentially damaging to macromolecules and cell structures; hence, their level should be tightly controlled; moreover, ROS imbalance acts as a signaling mechanism [21]. Prolonged/severe stress leads to a serious shift in redox balance and damage to macromolecules, known as oxidative stress [22]. To counteract oxidative stress, plants have elaborated a complex ROS scavenging system, including both enzymes and low molecular antioxidants. Superoxide anion is eliminated by superoxide dismutases (SOD, E.C.1.15.1.1) catalyzing its conversion to the less harmful H_2_O_2_ in different cell compartments [23]. Hydrogen peroxide is detoxified by several other enzymes. Catalases (CAT, E.C.1.11.1.6) operate in peroxisomes and glyoxisomes at high H_2_O_2_ concentrations and decompose H_2_O_2_ in a reaction where peroxide acts both as an acceptor and a donor of hydrogen [24]. Lower concentrations of H_2_O_2_ in the cytosol, chloroplasts, and mitochondria are scavenged by the enzymes and metabolites of the ascorbate–glutathione cycle, an essential part of which is the enzyme glutathione reductase (GR, E.C.1.6.4.2) which maintains the glutathione pool in a reduced state. Glutathione-S-transferase (GST, E.C.2.5.1.18) enzymes in plants have multiple functions, including the glutathione-dependent reduction in organic hydroperoxides that are formed during oxidative stress [21]. Various peroxidases (POX, E.C.1.11.1.17) also protect cells, decomposing H_2_O_2_ by oxidizing phenolic and endiolic co-substrates, operating in the cytosol, cell wall, and apoplast [25]. The concerted action of SOD with other hydroperoxide-scavenging enzymes and low-molecular antioxidants leads to efficient protection against oxidative stress.

Plant strategies for coping with drought are described as drought escape, drought avoidance, and drought tolerance; moreover, crops could use more than a single mechanism to withstand dehydration stress [26,27]. Drought escape consists of finishing the entire life cycle before extended stress events occur, for example, a hastened reproductive cycle and accelerated senescence. Drought avoidance includes morpho-physiological changes aimed at delaying the stress effects, such as deep rooting, strong stomatal sensitivity, reduced growth rate, leaf area reduction, osmotic adjustment, and moisture remobilization to the upper leaves and growing tips [28,29]. Drought tolerance relies on metabolic readjustments under already occurred water deficit, conserving the potential of the crop for recovery after stress [27]. Another classification of drought response divides plant species into water spenders (actively using water resources to support adaptive changes) and water savers (conserving water resources). According to numerous reports of cowpea response to drought stress, highlighting the increase in root biomass, small changes in leaf relative water content and the associated conserved membrane stability, reduced leaf area, and accumulation of osmolytes such as proline, this crop seems to rely mainly on drought avoidance mechanisms and water-saving strategies to cope with water deficit stress [30,31].

Drought stress response in crops, including cowpea, has a polygenic nature and strong effect of genotype by environment interaction [14], which seriously complicates breeding for drought tolerance. This highlights the importance of genetic diversity sources within a crop species for success, on one side, and the necessity for evaluating several morpho-physiological, biochemical, and molecular markers in screening for tolerance/sensitivity to osmotic stress, on the other side. The extensive use of elite, highly productive genotypes inevitably leads to diversity diminution; moreover, higher productivity could be at the expense of lower stress resistance [32,33]. Landraces, as local crop populations, naturally evolved in distinct geographical regions and, with conserved genetic diversity, are considered as a primary gene pool for crop improvement, especially in breeding for stress resilience [34]. Local crop populations are well-adapted to edaphic and climatic regional conditions and have higher yield stability due to certain resistance to locally spread diseases and pests. That is why local forms are a precious store of diversity for selection purposes in sustainable agriculture and a potential source of variation for drought resistance [7,12,15,35,36].

Local forms well adapted to particular agro-climatic conditions are useful germplasm resources which need to be characterized for stress tolerance. The aim of this study was to analyze the differences in the response to drought stress among local forms of cowpea, applying various morpho-physiological and biochemical parameters, and to highlight the mechanisms underlying cowpea drought tolerance/sensitivity at the vegetative growth stage. This comparative study was undertaken on five Bulgarian cowpea landraces, which had displayed differences in osmotic stress tolerance at the germination stage.

## 2. Results

### 2.1. General Response to Osmotic Stress in Cowpea Accessions at Vegetative Growth Stage

The accessions included in this study were selected by a preliminary test for tolerance to osmotic stress (15% *w*/*v* PEG 6000) at the germination stage and varied from tolerant to osmotic stress (B1E0103), intermediate tolerant (A4E0007, B1E0102), intermediate sensitive (A8E0542), to sensitive (BOE0034) at germination (Supplementary Table S1). Differences in tolerance to osmotic stress among accessions were expected to be also found at the autotrophic vegetative stage. Visible symptoms of stress, such as reduction in growth and leaf area and worsened habitus, were clearly observed after 2 weeks of treatment (Supplementary Figure S1) and are documented in Table 1. Leaf water deficit (WD) in PEG-treated plants presented a slight but significant increase (Table 1). Electrolyte leakage (EL%) as a membrane stability index had some tendency to increase under osmotic stress, but only in accession A8E0542 was a significant difference established compared to the respective control. The imposed osmotic stress diminished plant fresh weight (FW) and stem length, as well as significantly reduced the total leaf area in all accessions (Table 1).

The applied stress also diminished the quantity of newly formed leaves (Supplementary Table S2). In addition, control plants retained their simple leaves, whereas in PEG-stressed plants the first simple leaves underwent accelerated wilting and shedding. Total chlorophyll content and chl *a*/*b* ratio significantly diminished under stress in accessions A4E0007 and A8E0542, remained almost unchanged in accessions B1E0102 and B1E0103, and even increased in accession BOE0034; carotenoids were insignificantly changed under stress, and the chl to car ratio mostly reflected the changes in chlorophyll content (Supplementary Table S2).

Biochemical stress markers were studied in roots and in trifoliate leaves (Table 2). The total antioxidant activity had a tendency to increase in the roots of stressed plants. On the contrary, the tendency in leaves was towards a slight diminishing, except in BOE0034, where an increase had been observed (FRAP changes were significantly proven for this accession). MDA level as a marker of oxidative damage to lipids significantly increased only in roots of accession BOE0034. Proline content as a marker of osmotic stress intensity and ROS scavenger presented a significant increase in both roots and leaves of stressed cowpea plants, with the highest levels in the roots and leaves of accession A8E0542.

Phenols as secondary metabolites with scavenging properties contribute significantly to the total FRAP activity. In our study, a tendency to increase phenol content under stress was observed in the roots and leaves of stressed cowpea plants, which was significant for B1E0102 and BOE0034 in roots and for A4E0007, B1E0103, and BOE0034 in leaves, and was most expressed for accession BOE0034 (Table 3). Soluble sugars and starch had higher levels in roots in all stressed variants. Changes in soluble sugars in leaves under stress were insignificant, whereas starch content presented slight variations, significantly increasing under stress only in accession B1E0103.

Overall, the physiological and biochemical response of the studied cowpea accessions to osmotic stress revealed similar growth inhibition, leaf area diminution, relatively small water loss (increase in water deficit no more than 10%), consequently only slight membrane damage, no significantly increased MDA content as a marker of oxidative damage to membranes, but an increase in the content of protective metabolites such as proline and phenols. Roots presented more pronounced metabolic changes under stress than leaves, including increased total antioxidant activity, phenolic and carbohydrate content, and proline accumulation. Differences among accessions were mostly in leaf pigment content changes. The accession BOE0034, which was the most sensitive to osmotic stress at the germination stage, had a significant rise in MDA content in roots, the highest increase in total antioxidant capacity in roots, and was the only one with an increased FRAP content in leaves. Results indicate good control of the oxidative stress under the applied treatment, which could be due to mobilization of antioxidant enzymes.

### 2.2. Enzymatic ROS Scavenging System in Cowpea Under Osmotic Stress

#### 2.2.1. SOD Activity Changes

**Figure 1:**
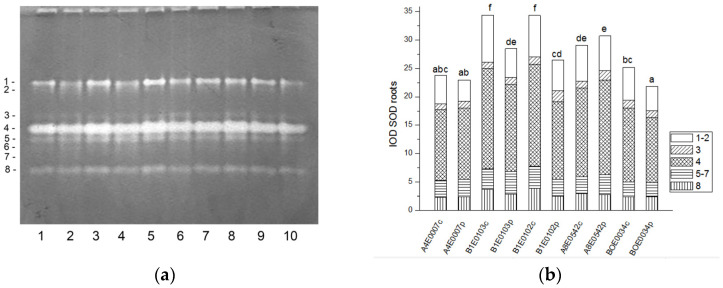
SOD isoform profile in cowpea leaves: (**a**) Activity staining of a representative gel, equal protein load of 30 µg per lane; isoforms are numbered on the left. Accessions and control/PEG treatment on lanes (**a**): 1—A4E0007 c, 2—A4E0007 p, 3—B1E0103 c, 4—B1E0103 p, 5—B1E0102 c, 6—B1E0102 p, 7—A8E0542 c, 8—A8E0542 p, 9—BOE0034 c, 10—BOE0034 p. (**b**) Integrated relative SOD activity in arbitrary cumulative units (IOD 1.10^3^) for the eight isoforms. Isoforms are with various patterns. Different letters above columns indicate statistically significant differences in SOD activity at the *p* 0.05 level among accessions/conditions.

**Figure 2:**
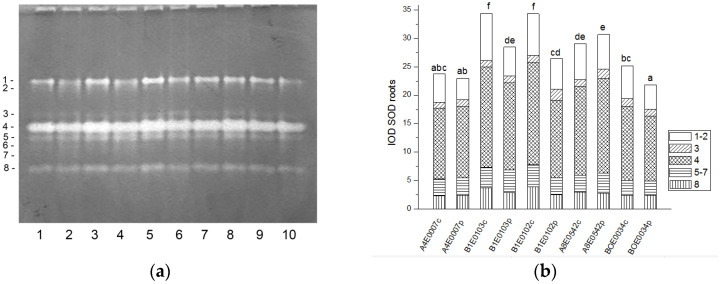
SOD isoform profile in cowpea roots: (**a**) Activity staining of a representative gel, equal protein load of 30 µg per lane; isoforms are numbered on the left. Accessions and control/PEG treatment on lanes (**a**): 1—A4E0007 c, 2—A4E0007 p, 3—B1E0103 c, 4—B1E0103 p, 5—B1E0102 c, 6—B1E0102 p, 7—A8E0542 c, 8—A8E0542 p, 9—BOE0034 c, 10—BOE0034 p. (**b**) Integrated relative SOD activity in arbitrary cumulative units (IOD 1.10^3^) for the eight isoforms. Isoforms are with various patterns. Different letters above columns indicate statistically significant differences in SOD activity at the *p* 0.05 level among accessions/conditions.

In the protein extracts from leaves as well as from roots of cowpea, eight bands of SOD activity (numbered in order of increasing electrophoretic mobility) were visible on the gels (Figure 1a and Figure 2a). Isoforms 4 and 7 had the highest share (about 77%) in the total leaf SOD activity, followed by isoform 1 (about 15% of the activity), while in roots the dominating isoform was 4, followed by 1 (53% and 20% of the total SOD activity, respectively). Isoform 3 was observed only in accession A8E0542.

A significant increase (from 122 to 131%, *p* ≤ 0.05) in the relative total SOD activity was observed in leaf extracts of osmotically stressed plants in all studied accessions (Figure 1b), which was mainly due to the activation of isoenzymes 4 and 5 (Figure 1a).

Unlike leaves, in the roots of stressed plants, SOD activity was significantly lower in accessions B1E0103, B1E0102, and BOE0034 and was the same as in controls for accessions A4E0007 and A8E0542 (Figure 2b). However, in accessions B1E0103 and B1E0102, isoenzymes 5 and 3, respectively, responded to osmotic stress with 12 and 46% increased activity (Figure 2a). Although the applied stress did not have a strong effect on the total activity of accessions A4E0007 and A8E0542, some specific SOD isoenzymes were up-regulated. In the case of the A8E0542 root sample, the isoenzymes 3 and 5 activities were increased by 31 and 17%, respectively. The activity of isoenzyme 3 was about 21% higher in the A4E0007 root sample compared to the control plants.

#### 2.2.2. CAT Activity Changes

**Figure 3:**
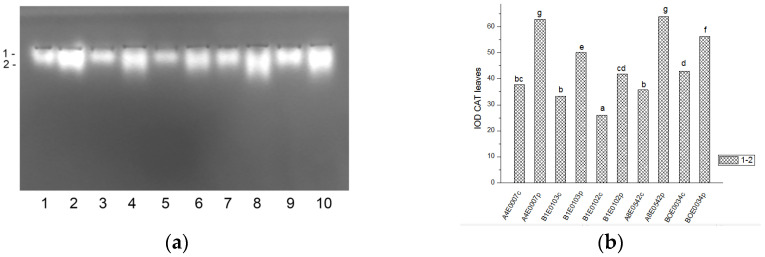
CAT activity pattern in cowpea leaves: (**a**) Activity staining of a representative gel, 10 µg protein load per lane; isoforms are numbered on the left. Accessions and control/PEG treatment on lanes (**a**): 1—A4E0007 c, 2—A4E0007 p, 3—B1E0103 c, 4—B1E0103 p, 5—B1E0102 c, 6—B1E0102 p, 7—A8E0542 c, 8—A8E0542 p, 9—BOE0034 c, 10—BOE0034 p. (**b**) Integrated relative CAT activity in arbitrary units (IOD 1.10^3^). Different letters above columns indicate statistically significant differences in CAT activity at the *p* 0.05 level among accessions/conditions.

**Figure 4:**
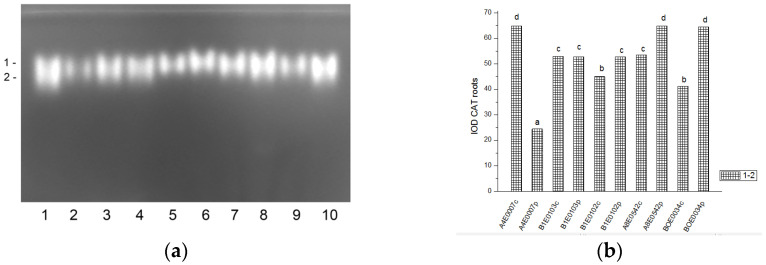
CAT activity pattern in cowpea roots: (**a**) Activity staining of a representative gel, 30 µg protein load per lane; isoforms are numbered on the left. Accessions and control/PEG treatment on lanes (**a**): 1—A4E0007 c, 2—A4E0007 p, 3—B1E0103 c, 4—B1E0103 p, 5—B1E0102 c, 6—B1E0102 p, 7—A8E0542 c, 8—A8E0542 p, 9—BOE0034 c, 10—BOE0034 p. (**b**) Integrated relative CAT activity in arbitrary units (IOD 1.10^3^). Different letters above columns indicate statistically significant differences in CAT activity at the *p* 0.05 level among accessions/conditions.

Staining for CAT revealed two not well separated activity bands in all *Vigna unguiculata* samples. In each sample, the intensity (activity) of isoenzyme 1 was almost equal to that of isoenzyme 2 (Figure 3a and Figure 4a).

The relative total CAT activity in leaves under osmotic stress was significantly higher than in their respective controls (by 67% for accession A4E0007, and by 51, 61, 79, and 31% for accessions B1E0103, B1E0102, A8E0542, and BOE0034, respectively) (Figure 3b). Compared to controls, total CAT activity in the roots of stressed plants varied depending on the accession. In A4E0007 it was 2.5 times lower, while in B1E0102, A8E0542, and BOE0034 it was 17, 21, and 56% higher. Only in accession B1E0103 was there no significant difference in CAT activity in roots from control and stressed plants. In general, the total CAT activity in leaves was higher than in roots (10 versus 30 µg of protein were applied to the wells of the respective gel).

#### 2.2.3. GR Activity Changes

**Figure 5:**
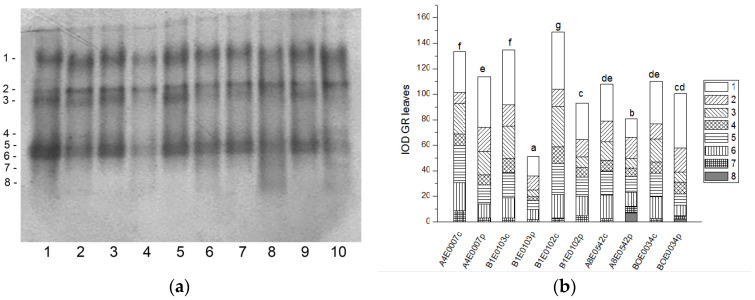
GR isoform profile in cowpea leaves: (**a**) Activity staining of a representative gel, 20 µg protein load per lane; isoforms are numbered on the left. Accessions and control/PEG treatment on lanes (**a**): 1—A4E0007 c, 2—A4E0007 p, 3—B1E0103 c, 4—B1E0103 p, 5—B1E0102 c, 6—B1E0102 p, 7—A8E0542 c, 8—A8E0542 p, 9—BOE0034 c, 10—BOE0034 p. (**b**) Integrated relative GR activity in arbitrary units (IOD). Isoforms are with various patterns. Different letters above columns indicate statistically significant differences in GR activity at the *p* 0.05 level among accessions/conditions.

The GR isoenzyme profile in the leaves of the five cowpea accessions was represented by eight common isoforms (Figure 5a). Isoform 8 appeared only in accessions A8E0542 and BOE0034 under stress. Isoform 1 accounted for 1/3 of leaf GR activity. Total GR activity was significantly suppressed as a result of applied osmotic stress in all samples except for accession BOE0034. In A4E0007, total leaf GR activity decreased to 85% of that of the control, but isoforms 1 and 2 activity was stimulated by 23 and 115%, respectively (Figure 5a,b). Although the total enzyme activity of B1E0102 and A8E0542 was 37% and 25% lower than the controls, their isoenzyme 7 was up-regulated (about 78 and 98% increase in activity, respectively). Osmotic stress had no significant effect on the total leaf GR activity of accession BOE0034; however, it led to a stimulation of the isoform 1 and 2 activities by 27 and 62%, respectively.

**Figure 6:**
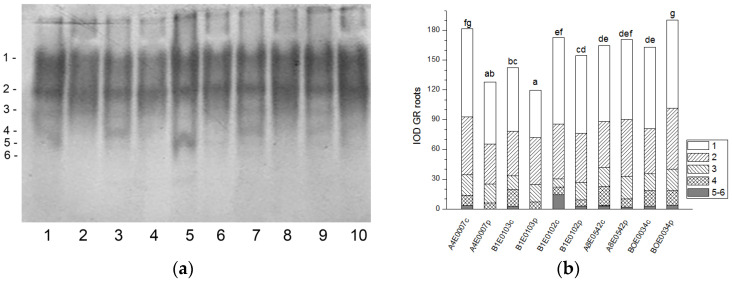
GR isoform profile in cowpea roots: (**a**) Activity staining of a representative gel, 20 µg protein load per lane; isoforms are numbered on the left. Accessions and control/PEG treatment on lanes (**a**): 1—A4E0007 c, 2—A4E0007 p, 3—B1E0103 c, 4—B1E0103 p, 5—B1E0102 c, 6—B1E0102 p, 7—A8E0542 c, 8—A8E0542 p, 9—BOE0034 c, 10—BOE0034 p. (**b**) Integrated relative GR activity in arbitrary units (IOD). Isoforms are with various patterns. Different letters above columns indicate statistically significant differences in GR activity at the *p* 0.05 level among accessions/conditions.

Six bands of GR activity were distinguished in all cowpea root samples (Figure 6a). The highest activity was registered for isoforms 1 and 2 (47 and 32% of the total GR activity, respectively). Isoform 5 was absent in accessions A4E0007 and B1E0103 under drought treatment. In the roots of stressed plants, the total GR activity increased by 17% (BOE0034), decreased by 29 and 16% (accessions A4E0007 and B1E0103, respectively) (*p* ≤ 0.05), or was not significantly different (A8E0542 and B1E0102) compared to their respective controls (Figure 6b). Despite these different responses, dehydration resulted in an increase in the activity of isoform 3 of accessions B1E0103, B1E0102, A8E0542, and BOE0034 by 29, 112, 18, and 29%, respectively, and of isoform 2 of A8E0542 and BOE0034 by 23 and 35%, respectively (Figure 6a).

#### 2.2.4. GST Activity Changes

**Figure 7:**
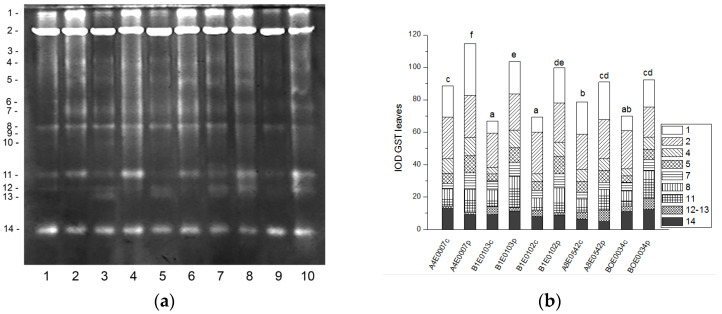
GST isoform profile in cowpea leaves: (**a**) Activity staining of a representative gel, 60 µg protein load per lane; isoforms are numbered on the left. Accessions and control/PEG treatment on lanes (**a**): 1—A4E0007 c, 2—A4E0007 p, 3—B1E0103 c, 4—B1E0103 p, 5—B1E0102 c, 6—B1E0102 p, 7—A8E0542 c, 8—A8E0542 p, 9—BOE0034 c, 10—BOE0034 p. (**b**) Integrated relative GST activity in arbitrary units (IOD 1.10^3^). Isoforms are with various patterns. Different letters above columns indicate statistically significant differences in GST activity at the *p* 0.05 level among accessions/conditions.

Fourteen GST isoenzymes were distinguished in the leaf samples of *Vigna unguiculata*(Figure 7a). Isoforms 1 and 2 accounted for 47% of the total activity, followed by isoform 14. The intensity of GST activity bands was significantly higher in the leaves of osmotically stressed plants of all tested accessions, resulting in significantly increased total GST activity compared to that of controls by 29, 55, 44, 15, and 32% in accessions A4E0007, B1E0103, B1E0102, A8E0542, and BOE0034, respectively (Figure 7b).

**Figure 8:**
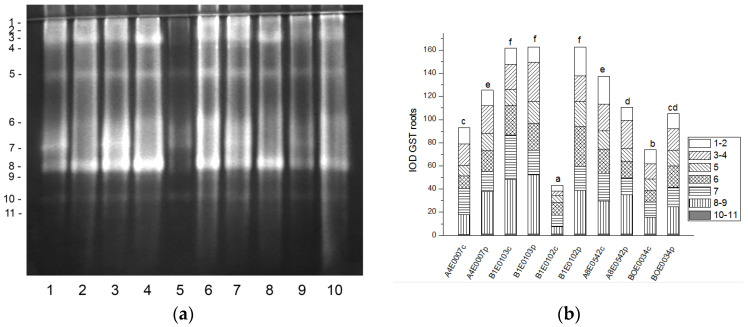
GST isoform profile in cowpea roots: (**a**) Activity staining of a representative gel, 20 µg protein load per lane; isoforms are numbered on the left. Accessions and control/PEG treatment on lanes (**a**): 1—A4E0007 c, 2—A4E0007 p, 3—B1E0103 c, 4—B1E0103 p, 5—B1E0102 c, 6—B1E0102 p, 7—A8E0542 c, 8—A8E0542 p, 9—BOE0034 c, 10—BOE0034 p. (**b**) Integrated relative GST activity in arbitrary units (IOD 1.10^3^). Isoforms are with various patterns. Different letters above columns indicate statistically significant differences in GST activity at the *p* 0.05 level among accessions/conditions.

Eleven GST isoforms were clearly visible in the root protein extracts, the intensity of which varied between the samples (Figure 8a). A significant increase (by 35, 275, and 42%, *p* ≤ 0.05) in the relative total GST activity was observed after osmotic stress in the roots of accessions A4E0007, B1E0102, and BOE0034 (Figure 8b). Their root isoenzymes 3 and 8 were the most responsive to the applied stress, showing the most strongly increased activity. Conversely, in accession A8E0542, total enzyme activity in stressed roots was 18% lower than the control, but the activity of isoform 8 was 21% higher. Although the total GST activity in the roots of dehydrated plants of accession B1E0103 was the same as the control, isoenzymes 3 and 5 were 59 and 37% more active, respectively (Figure 8a,b).

#### 2.2.5. POX Activity Changes

**Figure 9:**
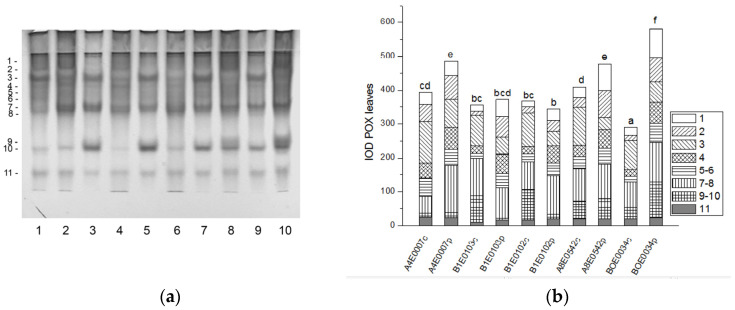
POX isoform profile in cowpea leaves: (**a**) Activity staining of a representative gel, 30 µg protein load per lane; isoforms are numbered on the left. Accessions and control/PEG treatment on lanes (**a**): 1—A4E0007 c, 2—A4E0007 p, 3—B1E0103 c, 4—B1E0103 p, 5—B1E0102 c, 6—B1E0102 p, 7—A8E0542 c, 8—A8E0542 p, 9—BOE0034 c, 10—BOE0034 p. (**b**) Integrated relative POX activity in arbitrary units (IOD). Isoforms are with various patterns. Different letters above columns indicate statistically significant differences in POX activity at the *p* 0.05 level among accessions/conditions.

The native PAGE of POX yielded eleven activity bands in cowpea leaves (Figure 9a).

A significant increase (by 23, 16, and 100%, respectively, *p* ≤ 0.05) in the relative total enzyme activity was observed after osmotic stress of accessions A4E0007, A8E0542, and BOE0034, due to upregulation of different isoenzymes (Figure 9a,b). The activity of POX isoenzymes 2, 4, 7, and 8 was significantly stimulated in A4E0007; isoenzymes 1, 2, 4, and 6 had increased activity in A8E0542, while in BOE0034 all, except isoenzyme 3, participated in the treatment response. Dehydration had no significant effect on the total POX activity of leaves of B1E0103 and B1E0102, but it led to increased activity of isoforms 1, 2, 4, and 6 in both accessions and of isoform 8 in B1E0102.

**Figure 10:**
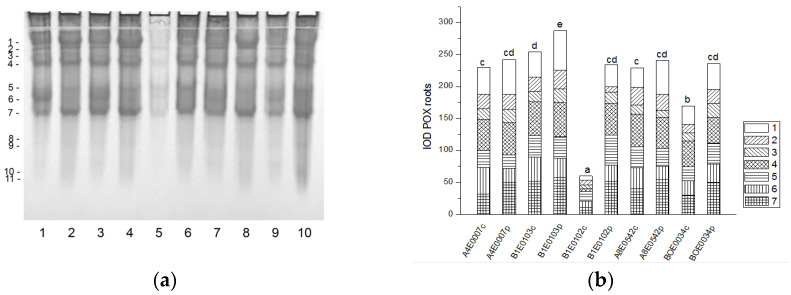
POX isoform profile in cowpea roots: (**a**) Activity staining of a representative gel, 30 µg protein load per lane; isoforms are numbered on the left. Accessions and control/PEG treatment on lanes (**a**): 1—A4E0007 c, 2—A4E0007 p, 3—B1E0103 c, 4—B1E0103 p, 5—B1E0102 c, 6—B1E0102 p, 7—A8E0542 c, 8—A8E0542 p, 9—BOE0034 c, 10—BOE0034 p. (**b**) Integrated relative POX activity in arbitrary units (IOD). Isoforms are with various patterns. Different letters above columns indicate statistically significant differences in POX activity at the *p* 0.05 level among accessions/conditions.

The POX isoenzyme profile of cowpea roots was represented by seven common isoforms. Four additional, fast-moving enzyme isoforms (8–11) were clearly visible only in the stressed roots of accession BOE0034 (Figure 10a). The total POX activity was significantly enhanced (by 232 and 39%, respectively) in the roots of osmotically stressed plants of accessions B1E0102 and BOE0034 compared to controls, with all enzyme isoforms responsible for the observed increase, albeit to varying degrees (Figure 10 a,b). In dehydrated roots of accessions A4E0007, B1E0103, and A8E0542, the total POX activity was not significantly different from that of the corresponding control. However, isoenzyme 1 was upregulated in those accessions (31, 55, and 79% increase, respectively), and isoenzyme 7 activity in A4E0007 and A8E0542 samples was 59 and 36% higher than the control, respectively.

Overall, cowpea response to osmotic stress in leaves was linked to enhanced enzymatic antioxidative protection—activation of SOD, CAT, GST, and, in some accessions, POX, along with changes in certain specific isoforms. GR activity was generally diminishing under osmotic stress. In roots under osmotic stress, the total SOD activity diminished or was unchanged, and CAT and GR activity had different changes comparing accessions. Data support a concerted mobilization of the enzymatic antioxidative protection under osmotic stress, especially in cowpea leaves.

## 3. Discussion

Drought tolerance mechanisms in *Vigna unguiculata* have been studied at germination [12,37], at vegetative [38,39] and at reproductive [16,40] growth stages, comparing different genotypes, among which are cultivars and traditional local forms. Different experimental designs have been used for estimation of drought tolerance at autotrophic growth stages—PEG addition in the nutrient medium [41], controlled irrigation of soil cultures [8,15,39,42], and water withholding [30,43], with advantages and limitations in every method. At germination, PEG is often used to induce drought stress as a simple, fast, and cost-effective method allowing screening of a large number of genotypes, and considerable differences have been reported comparing germination percentage, germination rate, and vigor index of cowpea genotypes [12]. Could data on germination under stress be extrapolated to other growth stages? Germplasm screening for drought tolerance at germination and early growth stages denoted root length, vigor index, and proline content as the most consistent and informative parameters [12]. However, some differences could be expected, as germination and subsequent seedling establishment rely on heterotrophic growth using the seed reserves, whereas at the vegetative growth stage, the growth is autotrophic and dependent on photosynthesis, and during flowering and grain filling, there should be a strong influence of source-sink interactions. It has been reported that in cowpea subjected to drought stress during grain filling, the yield was maintained despite the dramatic decrease in photosynthetic rate, by active translocation of photoassimilates from source organs to the developing grains [16]. Parallel experiments comparing water stress in cowpea cultivars induced at vegetative and at reproductive growth stages reported yield reduction after water restriction at both growth stages; besides, when applied at the vegetative stage, drought stress delayed flowering and reduced the number of flowers and weight of pods [44]. Some studies pointed out the role of deep rooting, changes in root architecture, and root/shoot ratio to overcome the negative effects of water limitation; the investment in root development under drought conditions has been linked to a higher drought tolerance in cowpea [8,38,41,44]. Screening 58 cowpea genotypes for drought tolerance at germination revealed that vigor index differed significantly under stress—diminished, remained unchanged, or even increased in some highly tolerant genotypes under drought stress conditions [12]. Our preliminary tests ranged the studied local accessions from osmotic stress tolerant to sensitive in the following order: B1E0103, A4E0007, B1E0102, A8E0542, and BOE0034 on the basis of their relative stress tolerance for the indices germination %, germination rate, and vigor index. We used similar conditions and the same stress level (15% PEG 6000) to compare these genotypes for osmotic stress response at the vegetative growth stage.

The physiological and biochemical response of the studied cowpea accessions to PEG-induced stress at the vegetative growth stage was growth inhibition, leaf area diminution, relatively small water loss with an increase in water deficit of no more than 10%, only slight membrane damage (Table 1), and an increase in the content of protective metabolites such as proline and phenols. Tolerance to water deficit in cowpea has been associated with increased membrane stability and smaller leaf area, proline accumulation, and an increase in phenol content [14]. Metabolic changes under stress were more pronounced in roots than in leaves, including increased total antioxidant activity, phenolic and carbohydrate content, and proline accumulation (Table 2 and Table 3). A stronger biochemical response in roots compared to shoots has been reported for drought-stressed common vetch [45]. Roots are the plant organs which primarily suffer from soil water scarcity, and sense and transmit stress signals to other plant parts. Contrary to the aboveground plant part, whose growth is inhibited under stress, the moderate drought can induce the formation of longer and thinner roots with increased absorbing surface and activated metabolism [46,47,48]. Common and distinct metabolic pathways have been reported to be engaged in roots and shoots in response to drought stress in a coordinated manner [49]. Enhanced osmoprotection with allocation of high quantities of amino acids, especially proline, sugars, and phenolics into roots in cowpea subjected to drought stress was established by metabolomics analysis [50]. The compatible solute proline acts additionally as a chaperone, redox buffer, and ROS scavenger, thus protecting membranes and proteins in stressed plants [51]. The accumulation of proline in leaves of drought-stressed cowpea has been positively correlated with stress severity and negatively with oxidative damage to membranes [43]. Consistent with proline’s protective role, a non-uniform distribution of this osmolyte was established with preferential proline accumulation in the upper part of osmotically stressed cowpea plants [52]. The total antioxidant activity measured by the FRAP method is informative for the mobilization of different low-molecular antioxidants such as ascorbic acid, phenols, flavonoids, and others. Plant phenols are aromatic compounds with benzene rings and one or more hydroxyl groups; as secondary metabolites, they have multiple functions. Phenols play an important role in the biosynthesis of pigments and lignin and cell wall strengthening, in plant protection against pests and pathogens, as well as in ROS scavenging [53]. Total antioxidant activity is well connected with the presence of phenolic compounds in vegetables and fruits [54]. In cowpea, increased polyphenolic compounds and antioxidant capacity have been reported under drought stress [42], and better stress tolerance was linked to more phenol accumulation under severe stress conditions [55].

Tolerance to water deficit in cowpea has been linked to increased membrane stability, diminished stomata opening and transpiration, and smaller leaf area. According to [31], shoot height, number of leaves, and leaf area were particularly sensitive morphological indicators for cowpea water deficit tolerance at the vegetative growth stage, along with accumulation of organic solutes and antioxidant enzymatic responses. In our study, cowpea response to osmotic stress in leaves was linked to enhanced enzymatic antioxidative protection. The antioxidant system plays a major role in drought tolerance [56]. A concerted higher activity of SOD, CAT, GST, and—in some accessions—POX has been observed, along with changes in certain specific enzyme isoforms (Figure 1, Figure 3, Figure 7 and Figure 9). In roots under osmotic stress, the total SOD activity diminished or was unchanged (Figure 2), while CAT and GR activity had different changes comparing accessions (Figure 4 and Figure 6). Antioxidative enzyme protection, mainly SOD, CAT, and POX, has been included in a number of studies on drought stress in cowpea [40,43,57,58,59,60]. Generally, increased leaf SOD [40,43,59], CAT [40,43,53,59,61], and POX [40,58,61] activities were reported in leaves of drought-stressed cowpea plants. However, according to [43], differences between genotypes could not be evaluated on the basis of antioxidant enzyme activities. For other legume species such as *Medicago sativa* L. and *Pisum sativum* L., the genotypes more tolerant to osmotic stress presented significantly higher mobilization of the antioxidative protection (enzyme activities and gene expression) compared to the sensitive genotypes [62,63].

SODs constitute the first line of defense against ROS, detoxifying the superoxide anions generated during photosynthesis and respiration, and the increase in both SOD and CAT activities in leaves may indicate activation of the processes of photorespiration during water stress. SOD also acts in cooperation with the enzymes detoxifying hydrogen peroxide. Interestingly, a reverse association between CAT and SOD with GR activities has been reported in drought-stressed common vetch [45], probably related to different functions of the enzymes. CAT detoxifies the bulk of H_2_O_2_ generated in photorespiration and fatty acid beta-oxidation, whereas the main function of GR is to maintain the glutathione pool in a reduced state in the ascorbate-glutathione cycle [64,65]. In our study, GR activity in leaves was generally diminished under osmotic stress (Figure 5). The enzymes of the ascorbate-glutathione cycle, ascorbate peroxidase and GR, have been reported to present higher activities with an increase in water stress intensity [43]. An upregulation of GR expression in a drought-susceptible cowpea cultivar and no expression change in a tolerant one have been established under progressive water stress, with a marked increase in recovery from stress [66,67], in concert with the role of GR in adjusting cellular redox balance by keeping the glutathione pool in reduced form [66]. Little is known about the role of GST in the oxidative protection of cowpea under drought stress except the finding that GST was among cowpea genes conferring drought tolerance [68]. In other legumes such as soybeans, GST and GR proteins have been reported to increase in roots under PEG-induced drought stress, with dynamic changes in different isoforms [69]. Six GST genes were upregulated under severe drought stress, and some GST genes were downregulated under light or moderate drought in a sensitive soybean cultivar at the flowering stage [70]. Overexpression of a wild soybean GST gene conferred osmotic stress tolerance in transgenic tobacco [71]. GR and GST are directly related to the metabolism of glutathione, which, except for ROS scavenging, is a precursor involved in other metabolic pathways such as the citric acid cycle and fatty acid synthesis [69]; therefore, the regulation of GR and GST seems to be complex and dependent on the stress level.

In our study, differences among accessions were mostly in leaf pigment content changes. The accession BOE0034, which was the most sensitive to osmotic stress at the germination stage (Supplementary Table S1), had a significant rise in MDA content in roots, the highest increase in total antioxidant capacity in roots, and was the only one with an increased FRAP content in leaves. On the contrary, the accession B1E0103, which presented the best relative stress tolerance at germination, did not have significantly increased MDA and total antioxidant capacity in either roots or leaves. B1E0103 had significantly higher overall SOD, POX, and GST activities in roots compared to BOE0034, a stable CAT activity pattern, and stress-induced decrease in GR activity, whereas in the roots of BOE0034, GR activity increased under stress. In leaves of stressed plants, the highest diminution in GR activity was registered for accession B1E0103, which was relatively higher in the controls, whereas in BOE0034, leaf GR activity was without significant changes under drought. These activity changes in ROS enzymes could reflect fluctuations for adjustment of the redox balance as well as better antioxidant protection in B1E0103.

## 4. Materials and Methods

### 4.1. Plant Material, Growth and Stress Conditions

Five local cowpea accessions (B1E0103, A4E0007, B1E0102, A8E0542, and BOE0034) originated from different locations in southern Bulgaria and were stored in the gene bank of the Institute of Plant Genetic resources (IPGR), Sadovo, and were included in this study. Seeds were surface sterilized for 3 min with 12% sodium hypochlorite, washed abundantly with distilled water (H_2_Od) and put to germinate on filter paper in Petri dishes with H_2_Od in the dark at room temperature (RT) for two days. Germinated seeds were transferred into pots (diameter of 9 cm and height of 7 cm) filled with perlite, five seedlings per pot, and grown in Hoagland nutrient solution (gradually increasing in strength from ⅛ to ½ and refreshed every other day) in a vegetation chamber under controlled conditions (16/8 h photoperiod, 150 μmol.m^−2^ s^−1^ light intensity, 24 °C and 60% air humidity). When plants were 16 days old with two expanding trifoliate leaves, half of the pots continued to receive ½ Hoagland solution (controls), and the other half was given 15% *w*/*v* PEG 6000 in ½ Hoagland solution (stress treatment) for an additional 16 days under the same growth conditions. At the end of the experiment, growth parameters, leaf water status, and electrolyte leakage were estimated on fresh plant material. For biochemical analyses, material was collected from roots and first-second trifoliate leaves, immediately frozen in liquid nitrogen, and stored at −80 °C until used.

### 4.2. Growth Parameters, Leaf Water Status, Membrane Stability, and Leaf Pigment Content

Biomass accumulation in belowground and aboveground plant parts was estimated gravimetrically on five individual plants for each condition, using an analytical balance; stem length was measured with a millimeter ruler; total leaf area was estimated after taking photos of all leaves on a plant and image analysis (ImageJ 1.54p software). Leaf water deficit (WD) was measured in triplicate on leaf segments from first trifoliate leaves (0.5 g), using the formula

WD% = (TW − FW)/TW × 100,

where FW—fresh weight, TW—weight of the same leaf material at full turgidity (after floating for 4–5 h at RT in 20 mL distilled water). Relative electrolyte leakage (EL%) from the same leaf material was determined as follows: conductivity of the electrolytes leaked in the water at full turgidity (initial conductivity); boiling leaves for 10 min and cooling down; conductivity measuring (total conductivity); and calculation of the ratio of initial to total conductivity. Measurements for WD and EL were in triplicate. Changes in leaf chlorophyll and carotenoid content were analyzed according to Arnon (1949) in 80% ethanol extracts from first trifoliate leaves (0.1 g) as described [72] in 96-well microplates. Optical density was registered at 470, 649, and 664 nm. Calculations were as follows: chlorophyll *a* (Chl *a*) (μg/mL) = 13.36 A_664_ − 5.19 A_649_; chlorophyll *b* (Chl *b*) (μg/mL) = 27.43 A_649_ − 8.12 A_664_ and carotenoids (μg/mL) = (1.000 A_470_ − 2.13 Chl *a* − 97.63 Chl*b*)/209.

### 4.3. Oxidative Stress Markers and Stress Responsive Metabolites

Total antioxidant activity was determined by the FRAP (Ferric reducing antioxidant power) assay [73] in 80% ethanol (*v*/*v*) extracts from roots and leaves (100 mg in 1.4 mL). The method is based on the reduction of a ferric tripyridyltriazine complex to its ferrous-colored form in the presence of antioxidants. Aliquots of 50 μL sample were mixed with 2 mL FRAP reagent, and, after incubation at 37 °C for 10 min, the absorbance was read spectrophotometrically at 593 nm. The values were expressed as the ferric reducing ability equivalent to that of 1 mmol/L FeSO_4_.

For malondialdehyde (MDA) and proline content estimation, plant material (0.5 g leaves, 1 g roots) was homogenized with 5 mL 0.1% (*w*/*v*) trichloroacetic acid on ice. Free proline was derivatized with acid ninhydrin, and absorbance was read at 520 nm [74]. MDA content was determined as thiobarbituric acid reactive substances [75].

Phenols, soluble sugar, and starch content were estimated in 80% of ethanol extracts of roots (0.2 g) and first-second trifoliate leaves (0.1 g) by spectrophotometric methods following the procedure in [72]. Folin–Ciocalteu reagent was applied for determination of phenolic substances [76] using caffeic acid for the standard curve; results are expressed as caffeic acid equivalents. Anthrone reagent and standard curves with glucose were used for the analysis of soluble sugar content in the supernatant and starch in the pellet [77].

### 4.4. Protein Extraction, PAGE, and Antioxidant Enzyme Activity Staining

Plant material (0.5 g from first and second trifoliate leaves and 1 g from roots) was ground in a mortar to a fine powder with liquid nitrogen. Protein extraction was performed in an ice bath. Homogenization was in 2 mL of 0.1 M potassium phosphate buffer (PPB) containing 5 mM dithiothreitol and 1 mM Na_2_EDTA; 20 mg Polyclar AT and 20 µL phenylmethylsulfonyl fluoride (from 0.1 M stock in DMSO) were added per sample during extraction. After sonication and centrifugation at 12,000× *g* for 30 min at 4 °C, the protein content in the supernatant was determined by the dye-binding assay [78]. The protein samples were subjected to discontinuous vertical Laemmli PAGE [79], but under non-denaturing and non-reducing conditions at 4 °C and a constant current of 35 mA per gel. When the dye front reached the bottom of the gel, individual gels were stained for the activities of superoxide dismutase (SOD), catalase (CAT), glutathione reductase (GR), glutathione S-transferase (GST), and unspecific peroxidases (POX).

To visualize the bands with SOD activity, 10% polyacrylamide gels (PAAG) loaded with 30 µg of protein from each sample were soaked in 0.1 mM nitroblue tetrazolium (NBT), 0.05 mM riboflavin, 1 mM Na_2_EDTA, and 0.3% (*v*/*v*) TEMED in 50 mM PPB (pH 7.8) for 20 min in the dark. Thereafter, the gels were submerged in H_2_Od and exposed to a light box for about 5 min to initiate the photochemical reaction [80]. For CAT activity staining, a method described in [81] was applied. The gels (8% PAAG) were pretreated in 0.1% H_2_O_2_ for 20 min and incubated in a staining solution containing 1% (*w*/*v*) ferric chloride and 1% (*w*/*v*) potassium ferricyanide mixed in equal volumes during use. Equal amounts of protein from the leaves (10 µg) or roots (30 µg) of the plants were applied to the wells of the respective gel. POX activity was resolved on 7% PAAG and visualized with benzidine, according to Ornstein [82]. Equal amounts of protein (50 µg) were applied to the wells. The staining solution for GR isoenzyme pattern and activity determination consisted of 0.24 mM 3-(4,5-dimethylthiazol-2-yl)-2,5-diphenyltetrazolium bromide (MTT), 0.34 mM 2,6-dichlorophenol indophenol, 3.6 mM GSSG, and 0.4 mM NADPH in 250 mM Tris-HCl buffer (pH 7.8). Gels (10%) with resolved protein samples (20 µg on each lane) were immersed in this solution for about 1 h in darkness [83]. GST isoforms and activities were detected as described [84]. Briefly, the 8% polyacrylamide gels, equilibrated in 100 mM PPB (pH 6.5) for 10 min, were transferred to a reaction mixture composed of 4.5 mM GSH, 1 mM 1-chloro-2,4-dinitrobenzene (CDNB), and 1 mM NBT in the same PPB buffer at 37 °C for 10 min. Further, the gels were incubated at room temperature in 100 mM Tris-HCl (pH 9.6) containing 3 mM phenazine methosulfate (PMS) until they became uniformly blue, while the bands with GST activity were achromatic.

After staining, the enzyme patterns were documented using the UVItec gel documentation system (Cambridge, UK) and analyzed using Gel-Pro32 Analyzer software (Media Cybernetics, Rockville, MD, USA). The intensity (activity) of each resolved band (isoenzyme) was measured as total integrated optical density (IOD) in arbitrary units. Each enzyme had more than one isoenzyme, and the sum of their IOD values was considered total enzyme activity for a particular treatment. The assay to determine the pattern and activity of each enzyme was repeated thrice. For easier comparison, the values for total enzyme activity and for the activity of the individual isoforms of each enzyme of the control plants were tentatively assumed to be 100%, and the values of the stressed plants were calculated relative to those of the corresponding control within the accession.

### 4.5. Statistics

Measurements were performed in triplicate, and values are presented as means and standard deviations. Statistically significant differences between all variants were estimated by the ANOVA multiple range test (Stat Graphics Plus2.1.) at significance *p* < 0.05.

## Figures and Tables

**Table 1:** Water status, membrane stability, and growth parameters in control (c) and PEG-stressed (p) cowpea plants.

Accession/ Condition	Leaf WD%	Leaf EL%	Leaf Area (cm^2^ Plant^−1^)	Plant FW (g Plant^−1^)	Stem L (cm Plant^−1^)
A4E0007 c	5.62 ^ab^	3.07 ^a^	153.68 ^c^	5.95 ^ab^	24.5 ^ab^
A4E0007 p	9.48 ^bc^	9.07 ^ab^	86.15 ^ab^	2.42 ^a^	14.3 ^a^
B1E0103 c	5.60 ^ab^	3.69 ^a^	168.45 ^cd^	5.65 ^ab^	18.2 ^ab^
B1E0103 p	14.12 ^cd^	5.13 ^a^	71.32 ^ab^	2.74 ^ab^	10.1 ^a^
B1E0102 c	7.02 ^ab^	2.66 ^a^	198.39 ^e^	6.06 ^ab^	14.4 ^ab^
B1E0102 p	18.13 ^d^	5.45 ^a^	52.63 ^a^	1.53 ^a^	7.9 ^a^
A8E0542 c	8.23 ^ab^	3.66 ^a^	164.04 ^cd^	4.64 ^ab^	18.4 ^ab^
A8E0542 p	18.13 ^d^	14.2 ^b^	60.86 ^ab^	1.54 ^a^	9.2 ^a^
BOE0034 c	4.42 ^a^	3.80 ^a^	184.76 ^cd^	6.39 ^b^	24.7 ^b^
BOE0034 p	16.99 ^d^	3.52 ^a^	95.08 ^b^	2.7 ^ab^	13.0 ^a^

WD—water deficit, EL—electrolyte leakage, FW—fresh weight, L—length. Values are mean of at least 3 independent replicates. Different superscript letters denote statistically significant differences at *p* 0.05 level.

**Table 2:** Stress markers in roots and leaves of control (c) and stressed (p) cowpea plants.

Accession/ Condition	FRAP (Fe eq g^−1^ FW)		MDA (nmol g^−1^ FW)		Proline (µg g^−1^ FW)	
	Roots	Leaves	Roots	Leaves	Roots	Leaves
A4E0007 c	14.32 ^ab^	106.7 ^a-d^	0.73 ^ab^	0.18 ^a^	20.42 ^a^	90.83 ^ab^
A4E0007 p	23.55 ^bc^	89.56 ^ab^	0.80 ^ab^	0.21 ^a^	94.26 ^d^	114.0 ^bcd^
B1E0103 c	15.49 ^ab^	108.0 ^a-d^	0.63 ^a^	0.28 ^ab^	22.07 ^a^	97.67 ^ab^
B1E0103 p	30.29 ^bc^	98.15 ^a-d^	0.79 ^ab^	0.18 ^a^	45.30 ^b^	130.04 ^cd^
B1E0102 c	9.81 ^a^	74.71 ^a^	0.75 ^ab^	0.12 ^a^	20.76 ^a^	93.32 ^ab^
B1E0102 p	18.19 ^abc^	94.59 ^ab^	0.75 ^ab^	0.21 ^a^	57.21 ^c^	106.52 ^bc^
A8E0542 c	27.75 ^bc^	134.9 ^d^	0.87 ^ab^	0.62 ^b^	25.51 ^a^	79.51 ^a^
A8E0542 p	39.18 ^cd^	108.7 ^cd^	0.99 ^b^	0.62 ^b^	132.88 ^e^	135.26 ^d^
BOE0034 c	18.48 ^abc^	76.96 ^ab^	0.84 ^ab^	0.15 ^a^	20.86 ^a^	95.72 ^ab^
BOE0034 p	58.53 ^d^	130.12 ^cd^	1.51 ^c^	0.34 ^ab^	48.99 ^b^	116.4 ^bcd^

Values are mean of at least 3 independent replicates. Different superscript letters denote statistically significant differences at *p* 0.05 level.

**Table 3:** Content of phenols, soluble sugars, and starch in roots and leaves of control (c) and PEG-stressed (p) cowpea plants.

Accession/ Condition	Phenols (mg CAE g^−1^ FW)		Soluble sugars (mg g^−1^ FW)		Starch (mg g^−1^ FW)	
	Roots	Leaves	Roots	Leaves	Roots	Leaves
A4E0007 c	1.51 ^ab^	3.626 ^ab^	7.88 ^a^	8.58 ^a^	3.99 ^ab^	23.24 ^d^
A4E0007 p	1.70 ^ab^	4.55 ^d^	10.22 ^abc^	10.17 ^ab^	5.53 ^b^	18.34 ^cd^
B1E0103 c	1.27 ^a^	3.54 ^a^	8.31 ^a^	10.13 ^ab^	3.98 ^ab^	8.96 ^ab^
B1E0103 p	1.99 ^ab^	4.07 ^bc^	12.17 ^bc^	8.87 ^a^	7.94 ^c^	18.55 ^cd^
B1E0102 c	1.30 ^a^	3.89 ^ab^	7.13 ^a^	8.75 ^a^	3.71 ^a^	7.84 ^a^
B1E0102 p	2.26 ^b^	4.19 ^bc^	13.11 ^c^	13.93 ^ab^	8.68 ^cd^	7.35 ^a^
A8E0542 c	1.14 ^a^	4.48 ^cd^	7.87 ^a^	11.67 ^ab^	3.26 ^a^	16.73 ^c^
A8E0542 p	1.73 ^ab^	5.04 ^d^	10.33 ^abc^	10.16 ^ab^	7.91 ^c^	14.91 ^bc^
BOE0034 c	1.45 ^ab^	3.81 ^a^	8.87 ^ab^	12.36 ^ab^	4.76 ^ab^	5.25 ^a^
BOE0034 p	3.38 ^c^	5.01 ^d^	12.94 ^c^	17.23 ^b^	9.63 ^d^	9.38 ^ab^

Values are mean of at least 3 independent replicates. Different superscript letters after values denote statistically significant difference at *p* 0.05 level.

## Data Availability

The original contributions presented in this study are included in the article/Supplementary Materials.

## Supplementary Materials

The following supporting information can be downloaded at: https://www.mdpi.com/article/10.3390/ijms26178352/s1.
