# Supplementary material for: Local Forms of Vigna unguiculata—Response to Osmotic Stress at Vegetative Growth Stage
Source: Int J Mol Sci. 2025 Aug 28;26(17):8352. doi: 10.3390/ijms26178352 (PMC12429160; doi:10.3390/ijms26178352)
Supplement: Supplementary file 1 [file ijms-26-08352-s001.zip › ijms-3771054-supplementary.pdf]

Table S1 Accessions studied and relative stress tolerance (RST) to 15% PEG 6000 at germination stage

accession	origin	RST G%	RST GR	RST VI	
**B1E0103**	Pazardjik	0,963	0,723	0,354	**tolerant**
**A4E0007**	Svilengrad	0,770	0,609	0,145	tolerant/intermediate
**B1E0102**	Petritch	0,695	0,477	0,245	intermrdiate
**A8E0542**	Kap. Andreevo	0,691	0,423	0,086	intermediate/sensitive
**BOE0034**	Haskovo	0,524	0,311	0,142	**sensitive**
**Mean±sd**		0,839-0,619	0,635-0,383	0,278-0,110	
mean		0,729	0,509	0,194	
sd		0,110	0,126	0,084	

G% - germination percentage; GR – germination rate; VI – seedling vigor index

Table S2. Number of trifoliate leaves and photosynthetic pigments content in control (c) and PEG stressed (p) cowpea plants.

**Accesion/ condition**	**Number 3-fol.leaves**	**Chl a+b ($mg.g^{-1}FW$)**	**Carotenoids ($mg.g^{-1}FW$)**	**Chl *a/b* ratio**	**Chls/car ratio**
**A4E0007 c**	3.4 ± 0.5^{bcd}	2.466 ± 0.155^{b}	0.447 ± 0.024^{f}	1.346^{b}	11.023^{b}
**A4E0007 p**	2.6 ± 0.5^{ab}	1.701 ± 0.076^{a}	0.397 ± 0.004^{ef}	0.819^{a}	8.569^{a}
**B1E0103 c**	3.8 ± 0.9^{cd}	2.488 ± 0.073^{b}	0.277 ± 0.004^{ab}	2.243^{f}	17.953^{f}
**B1E0103 p**	2.8 ± 0.4^{abc}	2.735 ± 0.102^{bc}	0.306 ± 0.002^{abc}	2.146^{e}	17.883^{f}
**B1E0102 c**	3.5 ± 0.8^{bcd}	2.394 ± 0.098^{b}	0.303 ± 0.012^{abc}	1.944^{d}	15.779^{d}
**B1E0102 p**	2.8 ± 0.8^{abc}	2.308 ± 0.348^{b}	0.260 ± 0.041^{a}	2.133^{e}	17.776^{f}
**A8E0542 c**	3.0 ± 0^{abcd}	3.170 ± 0.152^{c}	0.343 ± 0.001^{cde}	2.261^{f}	18.486^{g}
**A8E0542 p**	2.3 ± 0.4^{a}	2.497 ± 0.105^{b}	0.296 ± 0.013^{abc}	1.937^{d}	16.851^{e}
**BOE0034 c**	4 ± 0.8^{d}	2.489 ± 0.183^{b}	0.367 ± 0.027^{de}	1.871^{c}	13.550^{c}
**BOE0034 p**	3.8 ± 0.6^{cd}	3.173 ± 0.207^{c}	0.326 ± 0.024^{bcd}	2.130^{e}	19.448^{h}

Figure S1 Control and stressed plants – 15% PEG 6000 in the nutrient solution for 16 days at vegetative growth stage (developed trifoliate leaves)

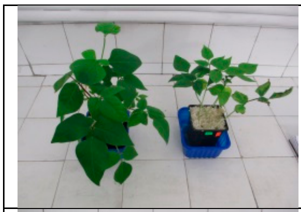	A4E0007
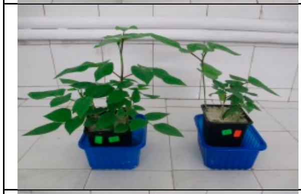	B1E0103
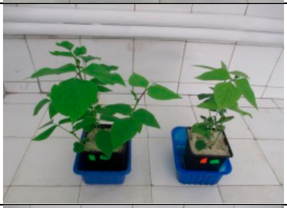	B1E0102
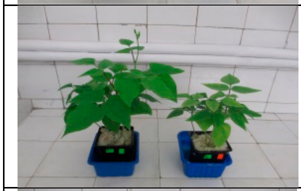	A8E0542
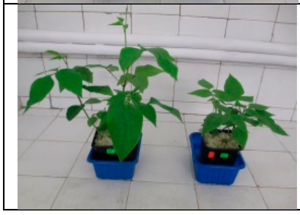	BOE0034
